# Changes in the incidence of invasive disease due to *Streptococcus pneumoniae, Haemophilus influenzae*, and *Neisseria meningitidis* during the COVID-19 pandemic in 26 countries and territories in the Invasive Respiratory Infection Surveillance Initiative: a prospective analysis of surveillance data

**DOI:** 10.1016/S2589-7500(21)00077-7

**Published:** 2021-05-24

**Authors:** Angela B Brueggemann, Melissa J Jansen van Rensburg, David Shaw, Noel D McCarthy, Keith A Jolley, Martin C J Maiden, Mark P G van der Linden, Zahin Amin-Chowdhury, Désirée E Bennett, Ray Borrow, Maria-Cristina C Brandileone, Karen Broughton, Ruth Campbell, Bin Cao, Carlo Casanova, Eun Hwa Choi, Yiu Wai Chu, Stephen A Clark, Heike Claus, Juliana Coelho, Mary Corcoran, Simon Cottrell, Robert J Cunney, Tine Dalby, Heather Davies, Linda de Gouveia, Ala-Eddine Deghmane, Walter Demczuk, Stefanie Desmet, Richard J Drew, Mignon du Plessis, Helga Erlendsdottir, Norman K Fry, Kurt Fuursted, Steve J Gray, Birgitta Henriques-Normark, Thomas Hale, Markus Hilty, Steen Hoffmann, Hilary Humphreys, Margaret Ip, Susanne Jacobsson, Jillian Johnston, Jana Kozakova, Karl G Kristinsson, Pavla Krizova, Alicja Kuch, Shamez N Ladhani, Thiên-Trí Lâm, Vera Lebedova, Laura Lindholm, David J Litt, Irene Martin, Delphine Martiny, Wesley Mattheus, Martha McElligott, Mary Meehan, Susan Meiring, Paula Mölling, Eva Morfeldt, Julie Morgan, Robert M Mulhall, Carmen Muñoz-Almagro, David R Murdoch, Joy Murphy, Martin Musilek, Alexandre Mzabi, Amaresh Perez-Argüello, Monique Perrin, Malorie Perry, Alba Redin, Richard Roberts, Maria Roberts, Assaf Rokney, Merav Ron, Kevin J Scott, Carmen L Sheppard, Lotta Siira, Anna Skoczyńska, Monica Sloan, Hans-Christian Slotved, Andrew J Smith, Joon Young Song, Muhamed-Kheir Taha, Maija Toropainen, Dominic Tsang, Anni Vainio, Nina M van Sorge, Emmanuelle Varon, Jiri Vlach, Ulrich Vogel, Sandra Vohrnova, Anne von Gottberg, Rosemeire C Zanella, Fei Zhou

**Affiliations:** a National Reference Centre for Streptococcus pneumoniae, University Hospitals Leuven, Leuven, Belgium; b Department of Microbiology, Immunology and Transplantation, KU Leuven, Leuven, Belgium; c National Reference Centre for Haemophilus influenzae, Laboratoires des Hôpitaux Universitaires de Bruxelles, Universitaire Laboratorium Brussel, Brussels, Belgium; d Faculté de Médecine et Pharmacie, Université de Mons, Mons, Belgium; e National Reference Centre for Neisseria meningitidis, Sciensano, Brussels, Belgium; f National Laboratory for Meningitis and Pneumococcal Infections, Center of Bacteriology, Institute Adolfo Lutz, São Paulo, Brazil; g National Microbiology Laboratory, Public Health Agency of Canada, Winnipeg, MB, Canada; h Department of Pulmonary and Critical Care Medicine, Center of Respiratory Medicine, China-Japan Friendship Hospital, Institute of Respiratory Medicine, Chinese Academy of Medical Sciences, National Clinical Research Center for Respiratory Diseases, Beijing, China; i National Reference Laboratory for Haemophilus Infections, Centre for Epidemiology and Microbiology, National Institute of Public Health, Prague, Czech Republic; j National Reference Laboratory for Meningococcal Infections, Centre for Epidemiology and Microbiology, National Institute of Public Health, Prague, Czech Republic; k National Reference Laboratory for Streptococcal Infections, Centre for Epidemiology and Microbiology, National Institute of Public Health, Prague, Czech Republic; l Department of Bacteria, Parasites and Fungi, Statens Serum Institut, Copenhagen, Denmark; m Nuffield Department of Population Health, Big Data Institute, University of Oxford, Oxford, UK; n Department of Zoology, University of Oxford, Oxford, UK; o Blavatnik School of Government, University of Oxford, Oxford, UK; p Immunisation and Countermeasures Division, National Infection Service, Public Health England, London, UK; q Respiratory and Vaccine Preventable Bacteria Reference Unit, National Infection Service, Public Health England, London, UK; r Meningococcal Reference Unit, National Infection Service, Public Health England, Manchester Royal Infirmary, Manchester, UK; s Trinity College Dublin, Dublin, Ireland; t Finnish Institute for Health and Welfare, Helsinki, Finland; u Institut Pasteur, Invasive Bacterial Infections Unit and National Reference Centre for Meningococci and Haemophilus influenzae, Paris, France; v Laboratory of Medical Biology and National Reference Centre for Pneumococci, Intercommunal Hospital of Créteil, Créteil, France; w Department of Medical Microbiology, German National Reference Center for Streptococci, University Hospital RWTH Aachen, Aachen, Germany; x German National Reference Center for Meningococci and Haemophilus influenzae, Institute for Hygiene and Microbiology, University of Würzburg, Würzburg, Germany; y Department of Health, Microbiology Division, Public Health Laboratory Services Branch, Centre for Health Protection, Hong Kong Special Administrative Region, China; z Department of Microbiology, The Chinese University of Hong Kong, Hong Kong Special Administrative Region, China; aa Department of Clinical Microbiology, Landspitali-The National University Hospital of Iceland, Reykjavik, Iceland; ab Irish Meningitis and Sepsis Reference Laboratory, Children's Health Ireland at Temple Street, Dublin, Ireland; ac Department of Clinical Microbiology, Beaumont Hospital, Dublin, Ireland; ad Department of Clinical Microbiology, Beaumont Hospital, Dublin, Ireland; ae Royal College of Surgeons in Ireland, Beaumont Hospital, Dublin, Ireland; af Government Central Laboratories, Ministry of Health, Jerusalem, Israel; ag Laboratoire National de Sante, Dudelange, Luxembourg; ah Department of Medical Microbiology and Infection Prevention, Amsterdam University Medical Center, University of Amsterdam, Amsterdam, Netherlands; ai Netherlands Reference Laboratory for Bacterial Meningitis, Amsterdam University Medical Center, University of Amsterdam, Amsterdam, Netherlands; aj Meningococcal Reference Laboratory, Institute of Environmental Science and Research Limited, Porirua, New Zealand; ak Streptococcal Reference Laboratory, Institute of Environmental Science and Research Limited, Porirua, New Zealand; al Department of Pathology and Biomedical Science, University of Otago, Christchurch, New Zealand; am Public Health Agency, Belfast, Northern Ireland; an National Reference Centre for Bacterial Meningitis, National Medicines Institute, Warsaw, Poland; ao Bacterial Respiratory Infection Service, Scottish Microbiology Reference Laboratories, Glasgow, UK; ap Centre for Respiratory Diseases and Meningitis, National Institute for Communicable Diseases, Division of the National Health Laboratory Service, Johannesburg, South Africa; aq Division of Public Health Surveillance and Response, National Institute for Communicable Diseases, Division of the National Health Laboratory Service, Johannesburg, South Africa; ar Department of Pediatrics, Seoul National University College of Medicine, Seoul, South Korea; as Department of Internal Medicine, Division of Infectious Diseases, Korea University Guro Hospital, Korea University College of Medicine, Seoul, South Korea; at Instituto de Recerca Pediatrica, Hospital Sant Joan de Deu, Barcelona, Spain; au Department of Microbiology, Tumor and Cell Biology, Karolinska Institutet, Stockholm, Sweden; av Department of Clinical Microbiology, Karolinska University Hospital, Stockholm, Sweden; aw Department of Laboratory Medicine, National Reference Laboratory for Neisseria meningitidis, Clinical Microbiology, Faculty of Medicine and Health, Örebro University, Örebro, Sweden; ax Public Health Agency of Sweden, Solna, Sweden; ay Swiss National Reference Centre for invasive Pneumococci, Institute for Infectious Diseases, University of Bern, Bern, Switzerland; az Public Health Wales, Cardiff, UK

## Abstract

**Background:**

*Streptococcus pneumoniae, Haemophilus influenzae*, and *Neisseria meningitidis*, which are typically transmitted via respiratory droplets, are leading causes of invasive diseases, including bacteraemic pneumonia and meningitis, and of secondary infections subsequent to post-viral respiratory disease. The aim of this study was to investigate the incidence of invasive disease due to these pathogens during the early months of the COVID-19 pandemic.

**Methods:**

In this prospective analysis of surveillance data, laboratories in 26 countries and territories across six continents submitted data on cases of invasive disease due to *S pneumoniae, H influenzae*, and *N meningitidis* from Jan 1, 2018, to May, 31, 2020, as part of the Invasive Respiratory Infection Surveillance (IRIS) Initiative. Numbers of weekly cases in 2020 were compared with corresponding data for 2018 and 2019. Data for invasive disease due to *Streptococcus agalactiae*, a non-respiratory pathogen, were collected from nine laboratories for comparison. The stringency of COVID-19 containment measures was quantified using the Oxford COVID-19 Government Response Tracker. Changes in population movements were assessed using Google COVID-19 Community Mobility Reports. Interrupted time-series modelling quantified changes in the incidence of invasive disease due to *S pneumoniae, H influenzae*, and *N meningitidis* in 2020 relative to when containment measures were imposed.

**Findings:**

27 laboratories from 26 countries and territories submitted data to the IRIS Initiative for *S pneumoniae* (62 837 total cases), 24 laboratories from 24 countries submitted data for *H influenzae* (7796 total cases), and 21 laboratories from 21 countries submitted data for *N meningitidis* (5877 total cases). All countries and territories had experienced a significant and sustained reduction in invasive diseases due to *S pneumoniae, H influenzae*, and *N meningitidis* in early 2020 (Jan 1 to May 31, 2020), coinciding with the introduction of COVID-19 containment measures in each country. By contrast, no significant changes in the incidence of invasive *S agalactiae* infections were observed. Similar trends were observed across most countries and territories despite differing stringency in COVID-19 control policies. The incidence of reported *S pneumoniae* infections decreased by 68% at 4 weeks (incidence rate ratio 0·32 [95% CI 0·27–0·37]) and 82% at 8 weeks (0·18 [0·14–0·23]) following the week in which significant changes in population movements were recorded.

**Interpretation:**

The introduction of COVID-19 containment policies and public information campaigns likely reduced transmission of *S pneumoniae, H influenzae*, and *N meningitidis*, leading to a significant reduction in life-threatening invasive diseases in many countries worldwide.

**Funding:**

Wellcome Trust (UK), Robert Koch Institute (Germany), Federal Ministry of Health (Germany), Pfizer, Merck, Health Protection Surveillance Centre (Ireland), SpID-Net project (Ireland), European Centre for Disease Prevention and Control (European Union), Horizon 2020 (European Commission), Ministry of Health (Poland), National Programme of Antibiotic Protection (Poland), Ministry of Science and Higher Education (Poland), Agencia de Salut Pública de Catalunya (Spain), Sant Joan de Deu Foundation (Spain), Knut and Alice Wallenberg Foundation (Sweden), Swedish Research Council (Sweden), Region Stockholm (Sweden), Federal Office of Public Health of Switzerland (Switzerland), and French Public Health Agency (France).

## Introduction

Invasive bacterial diseases, particularly bacteraemic pneumonia, meningitis, and sepsis, are leading causes of global morbidity and mortality among all age groups, especially among young children (aged <5 years), adolescents, and older adults (aged >65 years). The most common causes of these life-threatening diseases are *Streptococcus pneumoniae* (pneumococcus), *Haemophilus influenzae,* and *Neisseria meningitidis* (meningococcus), which normally reside in the nasopharynx or throat of healthy individuals and are transmitted person-to-person via the respiratory route.^1^, ^2^, ^3^, ^4^

In 2016, there were 336 million episodes of lower respiratory infections worldwide, leading to 2·4 million deaths.^5^ Respiratory infections were the sixth leading cause of death among all ages and the most common cause of death in children younger than 5 years.^5^ *S pneumoniae* was estimated to have caused 197 million episodes of pneumonia, which led to more than 1·1 million deaths worldwide—more deaths than the combined total number of pneumonia deaths due to *H influenzae* serotype b, influenza, or respiratory syncytial virus.^5^ Globally, the number of deaths due to meningitis among all ages was around 300 000 in 2016, from an estimated 2·8 million meningitis episodes.^6^ Meningitis outbreaks due to these three bacteria (*N meningitidis* in particular) have occurred worldwide.^5^, ^6^

SARS-CoV-2 is a novel coronavirus that was first recognised as a cause of respiratory infection in early 2020 and causes COVID-19 in humans. 83·6 million cases of COVID-19 and 1·8 million COVID-19-related deaths have been reported worldwide as of Dec 31, 2020.^7^ Viral respiratory infections are associated with an increased risk of subsequent bacterial infections, especially invasive diseases and pneumonia. For example, the high mortality of the 1918 influenza pandemic was strongly associated with post-viral pneumonia caused by *S pneumoniae* in the absence of antimicrobials to treat bacterial pneumonia.^8^, ^9^, ^10^, ^11^, ^12^ Therefore, there is potential for increased rates of invasive bacterial diseases subsequent to SARS-CoV-2 infection. Alternatively, containment measures initiated in many countries to reduce viral transmission could result in decreased invasive disease due to a concomitant reduction in transmission of respiratory-associated bacteria.

**Research in context**

**Evidence before this study**

We searched PubMed, *bioRxiv*, and *medRxiv* for articles published in English from database inception up to Dec 31, 2019, before the COVID-19 pandemic, using search terms “pandemic” AND “microbial transmission” OR “transmission” AND “containment”. 262 papers were identified, none of which met our inclusion criteria (ie, a study that described large-scale containment measures implemented during a pandemic that reduced the burden of disease due to pathogens other than SARS-CoV-2). Although strategies for containment and reducing transmission of an epidemic or pandemic pathogen have been well described in the literature, there is currently an evidence gap regarding the extent to which large-scale containment measures implemented during a pandemic reduce the burden of infectious diseases due to pathogens other than the one causing the pandemic.

**Added value of this study**

We used existing laboratory data to address the effect of COVID-19 and associated containment measures on the incidence of invasive diseases caused by *Streptococcus pneumoniae, Haemophilus influenzae*, and *Neisseria meningitidis*, which are all transmitted via the respiratory route, with *Streptococcus agalactiae* as a non-respiratory comparator micro-organism. We rapidly established an international network of laboratories in 26 countries and territories, compiled a large invasive disease dataset of more than 80 700 case records, analysed data for national policy decisions and containment measures in each country using the Oxford COVID-19 Government Response Tracker, and examined the movements of people during the early months of the COVID-19 pandemic using Google COVID-19 Community Mobility Reports. Our study showed that the incidence of invasive disease due to *S pneumoniae, H influenzae*, and *N meningitidis* declined sharply in all participating countries following the introduction of COVID-19 containment measures in early 2020, whereas the incidence of invasive disease due to *S agalactiae* did not. The decreases in incidence of these invasive diseases were largely consistent across countries, despite variation in the stringency of containment measures adopted in the early stages of the COVID-19 pandemic.

**Implications of all the available evidence**

High-quality, prospective microbiological disease surveillance is crucial to global health. COVID-19 containment measures reduce the transmission of respiratory pathogens and associated diseases, but they also impose a heavy burden on society that must be carefully considered. Invasive diseases due to *S pneumoniae, H influenzae*, and *N meningitidis* are among the leading causes of death and disability worldwide. Safe and effective vaccines for all three pathogens are used in many, but not all, countries and should be implemented more widely. Finally, the invasive disease burden is likely to increase as COVID-19 containment measures are relaxed. Therefore, ongoing microbiological surveillance, such as that shown in this study, is essential.

Given the severity of the diseases they cause, invasive infections (eg, meningitis and sepsis) are medical emergencies that are treated in hospital without delay. Clinical microbiology laboratories in many countries are required to report invasive infections due to *S pneumoniae, H influenzae*, and *N meningitidis* to national health authorities, and many also request referral of any isolates to reference laboratories for surveillance purposes. We established the Invasive Respiratory Infection Surveillance (IRIS) Initiative with a network of reference laboratories in 26 countries and territories to rapidly analyse laboratory-confirmed invasive bacterial infection data during the COVID-19 pandemic. We compared the incidence of invasive bacterial infection with *S pneumoniae, H influenzae*, and *N meningitidis* during the COVID-19 pandemic with rates in previous years.

## Methods

### Study design and participants

We approached microbiology laboratories with established invasive disease surveillance systems to join the IRIS network between April 30 and June 10, 2020. All IRIS laboratories were national reference laboratories except for the laboratories in Spain (representing the territory of Catalonia) and China (representing one institute in Beijing). Confirmed cases of invasive disease due to *S pneumoniae, H influenzae*, or *N meningitidis* (detected from a normally sterile site or from a patient with invasive disease) plus the sampling date were collected. All 26 countries or territories submitted data for *S pneumoniae,* and most countries or territories submitted data for *H influenzae* (24 laboratories) and *N meningitidis* (21 laboratories; appendix pp 2–3). Nine laboratories also submitted data for *Streptococcus agalactiae* (Lancefield group B streptococcus) as a control, non-respiratory pathogen. *S agalactiae* is often found in the healthy gastrointestinal and lower genital tract and is a risk factor for invasive disease in pregnant women and neonates (due to vertical transmission during childbirth);^13^ however, invasive disease due to *S agalactiae* is increasing among adults who are not pregnant, although the route of transmission is unclear.^14^ *S agalactiae* was used as the control because it is a non-respiratory pathogen and a notifiable invasive disease. Moreover, there were nine laboratories in IRIS that routinely collected data for *S agalactiae*, meaning this pathogen could be used as an indicator for whether surveillance was affected by the pandemic (eg, whether laboratories were not receiving specimens as expected because of disruptions caused by the pandemic).

### Data collection

No patient-identifiable data were collected. A private IRIS dataview was set up as part of the PubMLST suite of databases and IRIS participants were able to upload and view study data.^15^ A subset of the French *N meningitidis* dataset was published previously.^16^ Cases of *S pneumoniae, H influenzae, N meningitidis,* and *S agalactiae* diagnosed between Jan 1, 2018, and May 31, 2020, were analysed. Case counts were summed by the International Organization for Standardization (ISO) week.

The Oxford COVID-19 Government Response Tracker (OxCGRT) collects information on policies and interventions that governments have implemented during the COVID-19 pandemic.^17^ Data were collected from more than 180 countries for 18 different indicators, which were combined into composite indices that measured the magnitude of government responses. The stringency index provided a combined estimate of the stringency of public information campaigns plus containment measures, including school closures, workplace closures, cancellation of public events, restrictions on gatherings, closures of public transport, stay at home requirements, restrictions on internal population movement (eg, recommendations not to travel between regions or cities), and international travel controls. The OxCGRT dataset was downloaded on Oct 5, 2020, and the daily stringency index data were converted to a mean ISO week value and merged with the IRIS case data, with zero indicating least stringent and 100 indicating most stringent.

The Google COVID-19 Community Mobility Reports provide anonymised, aggregated within-country data for the movement of people by capturing mobile device location history data from Google users in six categories (ie, grocery and pharmacy, parks, transit stations, retail and recreation, residential, and workplaces). Data that could identify individuals are not available from Google CCMR. Daily mobility data were calculated as a percentage change from the Google CCMR baseline day, which was the median value from the 5-week period from Jan 3 to Feb 6, 2020. Google COVID-19 Community Mobility Reports will potentially underestimate mobility changes for countries that experienced widespread COVID-19 disruption during the baseline period; however, we expect that only East Asian countries and territories would be affected (ie, China, Hong Kong, and South Korea). Google COVID-19 Community Mobility Reports data were downloaded on Oct 5, 2020.

### Statistical analysis

Statistical evidence for the effect of COVID-19 containment measures on the weekly number of cases for each infection studied was assessed using generalised linear models. Analyses evaluated evidence for both step and linear slope changes in the levels of each infection (measured as cases per week) following the implementation of COVID-19 containment measures. These analyses were undertaken using combined data across countries and territories for *S pneumoniae, H influenzae*, and *N meningitidis*, limited to those countries and territories with data for all three pathogens. For *S pneumoniae*, in which numbers of cases were higher than for the other two pathogens, analyses used country-level data, with both country-specific analyses showing the pattern of effects across countries and combined analyses to evaluate the effect of interventions varying across countries in their intensity and timing (ie, school closures).

For *S pneumoniae*, models were first individually fitted to the dataset for each country using a Poisson distribution, which included a scaling factor to correct for overdispersion, and results summarised by meta-analysis. Second, models were fitted as a single mixed-effects negative binomial model. Country-specific interruption timepoints were based on Google COVID-19 Community Mobility Reports data for workplace mobility, selecting the week containing the midpoint of the decline in work-associated mobility (ie, the week containing the day halfway from baseline [maximum] to the new lower [minimum] value). No Google COVID-19 Community Mobility Reports data were available for Iceland or China. Therefore, we assigned Iceland the modal week of other European countries and we based China's interruption point (set at week 5) on news reports of policy decisions (appendix p 4).

To adjust for underlying secular trends and seasonality in each country, a linear term for long-term trends and Fourier series with two terms, allowing country-specific cyclical seasonal variations, were fitted to the model for each country with a regression equation (appendix p 5). Analyses produced incidence rate ratios, SEs, and 95% CIs for each country for the step change following the interruption point: the ratio of cases per week after the interruption point compared with the number of cases that occurred before interruption, and slope change for further ongoing decline from this timepoint.

For *S pneumoniae*, a meta-analysis of the estimates for each country was done to generate pooled effect sizes, using the inverse-variant fixed-effects and restricted maximum likelihood random-effects method. This meta-analysis assessed the effect of the step, the slope, and linear combinations of these, to estimate the change from expected numbers of *S pneumoniae* cases over 4-week and 8-week periods following the interruption timepoint.

A negative binomial-distribution mixed-effects model was used to evaluate evidence for the effects of specific interventions on weekly counts. This model included one sine and cosine term for seasonality (a second term was unsupported), a linear effect in time, and a step and slope for country-specific interruption time in the fixed-effects part of the model. Week numbers were edited for countries in the southern hemisphere (ie, running from weeks 27 to 52 and then weeks 1 to 26) to allow joint modelling of seasonality. Random effects were included for seasonality, step, and slope variables. Widespread school closures (ie, OxCGRT level 2 or 3) were used to model the effects of specific policy changes alongside general changes in behaviours indexed by the Google COVID-19 Community Mobility Reports workplace mobility data. A further country-specific interruption was modelled using the date of school closures and allowing both a step and slope change from that date. Models were individually checked for assumption violation. Sensitivity testing was done to assess whether a lag produced a better model fit.

Analyses were also run on the combined weekly count data for each of the four pathogens. These analyses included only countries and territories that had submitted data for all of *S pneumoniae, H influenzae*, and *N meningitidis* to allow comparability. A common interruption week (ie, week 11, when the COVID-19 pandemic was declared by WHO) was applied and step and slope parameters were estimated. A counterfactual estimate was calculated for the expectation of cases in the absence of an interruption to the time series. Statistical analyses were done in Stata version 16.1 and R version 3.6.1.

### Role of the funding source

The funders of the study had no role in study design, data collection, data analysis, data interpretation, or writing of the report.

## Results

**Figure 1:**
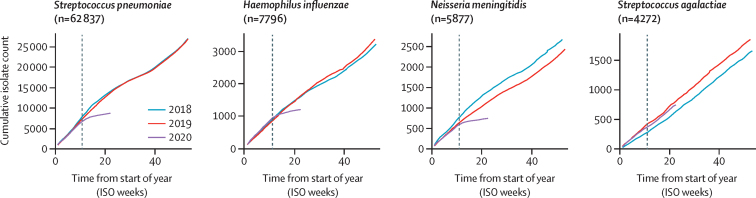
Cumulative number of invasive disease cases collected by Invasive Respiratory Infection Surveillance laboratories each week from Jan 1, 2018, to May 31, 2020 Data for *Streptococcus pneumoniae, Haemophilus influenzae*, and *Neisseria meningitidis* were obtained from Belgium, Brazil, Canada (*S pneumoniae* only), China (*S pneumoniae* and *H influenzae* only), Czech Republic, Denmark, England, Finland, France, Germany, Hong Kong, Iceland, Ireland, Israel (*S pneumoniae* and *H influenzae* only), Luxembourg, the Netherlands, New Zealand, Northern Ireland, Poland, Scotland, South Africa, South Korea (*S pneumoniae* and *H influenzae* only), Spain (*S pneumoniae* and *N meningitidis* only), Sweden, Switzerland (*S pneumoniae* and *H influenzae* only), and Wales. Data for *Streptococcus agalactiae* were obtained from Denmark, England, Finland, Germany, Iceland, Ireland, Israel, the Netherlands, and Poland. The grey dotted line (on week 11) shows when WHO officially declared the COVID-19 pandemic. ISO=International Organization for Standardization.

37 laboratories from 26 countries submitted invasive disease data to IRIS for one or more pathogens: *S pneumoniae* (62 837 cases from 26 countries); *H influenzae* (7796 cases from 24 countries); and *N meningitidis* (5877 cases from 21 countries) between Jan 1, 2018, and May 31, 2020. There was a substantial and sustained reduction in the number of invasive cases of *S pneumoniae, H influenzae*, and *N meningitidis* diagnosed between March and May, 2020, versus the previous 2 years (figure 1). This finding was in clear contrast to numbers in 2018 and 2019, when the overall numbers of *S pneumoniae* and *H influenzae* cases were very similar. A similar reduction was observed for *N meningitidis* in 2020, although the number of cases varied between 2018 (2700 cases) and 2019 (2457 cases). The reductions in the weeks following the interruption to the expected time series were strongly supported from likelihood ratio tests of models with and without parameters for the reduction (p<0·0001; appendix p 6).

One explanation for the reduction in numbers of invasive cases of *S pneumoniae, H influenzae*, and *N meningitidis* was that routine invasive disease surveillance was disrupted while countries were responding to COVID-19; however, IRIS laboratories did not observe significant disruptions in routine submissions of *S pneumoniae, H influenzae, N meningitidis*, or *S agalactiae* to the reference laboratories. To investigate the plausibility of this idea, we analysed 4272 cases of *S agalactiae* submitted over the same time period from nine IRIS laboratories in the same surveillance areas as for *S pneumoniae, H influenzae*, and *N meningitidis*. We found no evidence of any change in *S agalactiae* submissions in 2020 versus 2018 and 2019, supporting the view that the reductions in *S pneumoniae, H influenzae*, and *N meningitidis* cases in 2020 were consistent with decreases in disease incidence and not a consequence of disruptions in routine case reporting or isolate referral (figure 1).

**Figure 2:**
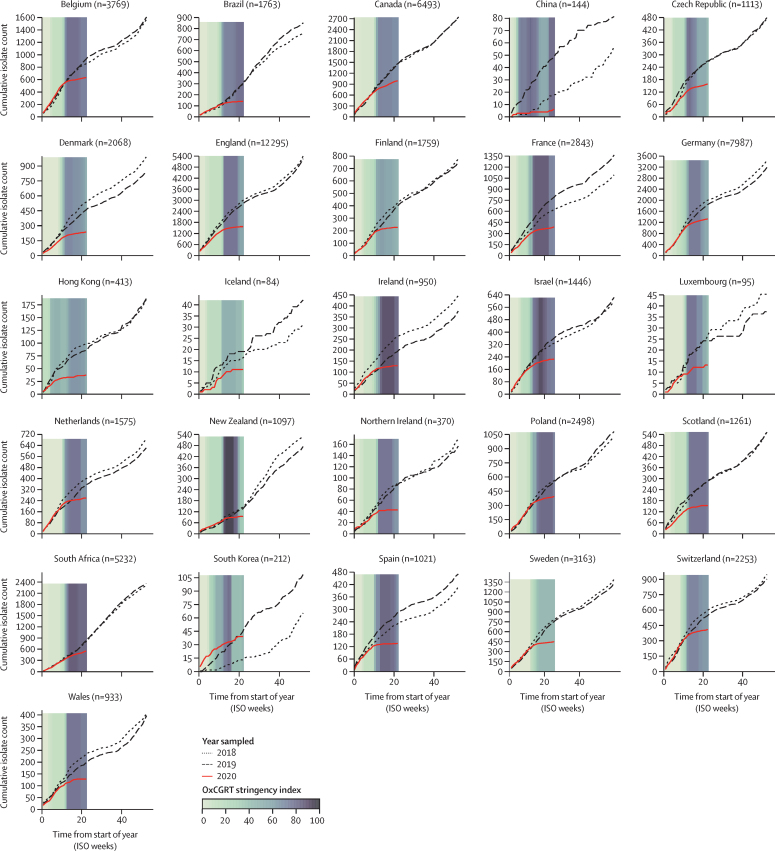
Annual invasive *Streptococcus pneumoniae* cases submitted to Invasive Respiratory Infection Surveillance laboratories in 26 countries and territories from Jan 1, 2018, to May 31, 2020 Coloured bars represent the mean weekly Oxford COVID-19 Government Response Tracker (OxCGRT) stringency index values on a scale from 0 to 100, with larger (darker) values indicating that higher stringency measures were enacted within a country. Data for South Korea were submitted from two surveillance networks, one of which started invasive disease surveillance in September, 2018, so data presented here for that hospital are only from September, 2018, onwards, whereas the data from the other hospital are from January, 2018, onwards. ISO=International Organization for Standardization.

To assess the effect of COVID-19 containment measures on the reduction in invasive *S pneumoniae, H influenzae*, and *N meningitidis* infections, the weekly bacterial case submission data were compared with the OxCGRT stringency index calculated for each country. WHO officially declared the COVID-19 pandemic in week 11 of 2020, by which point all countries and territories represented in IRIS had initiated some COVID-19 containment measures, ranging from 11 to 81 on the stringency index scale (figure 2). By week 15, all countries had rapidly increased containment measures and public information campaigns: 15 countries and territories had a stringency index score of 80 or more, eight (Brazil, Canada, China, Czech Republic, Denmark, Germany, Hong Kong, and Switzerland) had scores between 60 and 80, and three (Finland, Iceland, and Sweden) had scores between 45 and 60. China initiated stringent containment measures and public information campaigns in week 4, then reduced the measures slightly, and by week 20 increased to high stringency measures. Although the stringency of the imposed containment measures varied by country, there was a pronounced reduction in notifications of invasive disease due to *S pneumoniae* among all participating countries and this reduction was sustained up to the end of May, 2020. Similar trends were observed for *H influenzae* and *N meningitidis*, but not for *S agalactiae* (appendix pp 7–9).

To assess the extent to which societal choices also influenced the reductions in invasive disease, we compared the movement of people within each country using Google COVID-19 Community Mobility Reports data (appendix p 10). All 26 countries and territories, regardless of the stringency of the containment measures imposed by their governments, experienced a decrease in workplace visits and an increase in time spent in residential areas. The largest changes in movement (compared with baseline) occurred at the time WHO declared the COVID-19 pandemic and shortly thereafter. Countries that implemented the most stringent COVID-19 containment measures also had the largest changes in movements around workplaces and residences, but even countries with moderate containment measures (ie, Finland, Iceland, and Sweden) experienced large changes in population movements in the same direction as other countries. Changes in the movement of people within Hong Kong and South Korea were less variable and occurred earlier. Countries in the southern hemisphere (ie, Brazil, New Zealand, and South Africa) had different patterns of movement in the early weeks of 2020, in part because of summer holidays. By the end of May, 2020, the mobility data in many countries were shifting back to baseline levels and movements around workplaces were beginning to increase above baseline in several countries; however, Brazil, Canada, Ireland, South Africa, Spain, and the UK had not yet reverted back to baseline levels of movement for either workplaces or residences.

**Figure 3:**
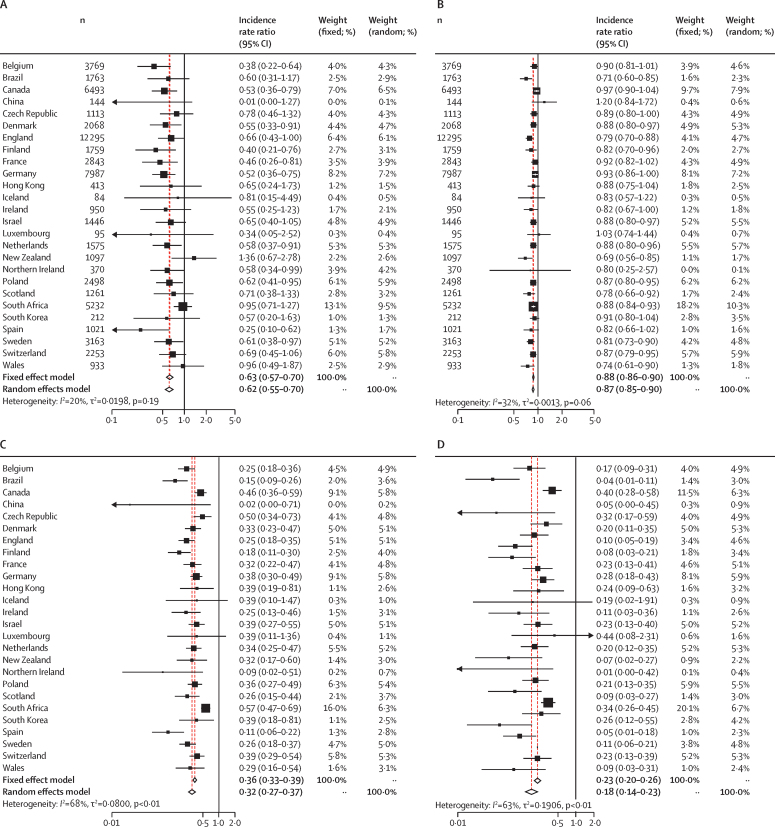
Estimated country-specific incidence rate ratios for invasive disease due to *Streptococcus pneumoniae* following interruptions in population mobility during the COVID-19 pandemic Estimated country-specific incidence rate ratios calculated using an interrupted time series model that allows for a step (A) or slope (B) reduction in invasive disease. Estimated country-specific incidence rate ratios after 4 weeks (C) or 8 weeks (D) from the point at which national mobility was significantly interrupted (appendix p 4).

62 837 cases of *S pneumoniae* were reported between Jan 1, 2018, and May 31, 2020 (ie, week 22 in 2020). Our models estimated that social changes caused by the COVID-19 pandemic led to a 38% decrease in the incidence of reported *S pneumoniae* invasive infections (incidence rate ratio [IRR] 0·62 [95% CI 0·55–0·70]) immediately following the interruption timepoint (measured by the step change parameter) followed by an additional 13% average weekly reduction up to the end of the study period (May 31, 2020; 0·87 [0·84–0·89]). Similar results were obtained from the combined model analysis for both step change (0·56 [0·49–0·65]) and slope (0·87 [0·84–0·90]) parameters and there was no strong evidence to favour a lag between movement changes and effects on infection rates. Although there was some variation among countries, the effect sizes were largely similar (figure 3). The deviation from expected numbers of invasive infections in the northern hemisphere versus the southern hemisphere did not follow an obvious pattern. That is, there was no evidence that latitude had a crucial role in the impact of COVID-19 and COVID-19 containment measures on the incidence of invasive infections.

Compared with expectations based on the time series for 2018 and 2019, these reductions translated into a decrease in the incidence of reported *S pneumoniae* infections of 68% at 4 weeks (IRR 0·32 [95% CI 0·27–0·37]) and 82% at 8 weeks (0·18 [0·14–0·23]) following the week when movement changes were noted. Adding country-specific terms for school closures into the combined model analysis did not substantially improve fit (p=0·09) with strong support remaining for a decrease following reduced mobility (as indexed by Google COVID-19 Community Mobility Reports data) and little evidence for additional effects linked specifically to school closures.

## Discussion

The IRIS Initiative rapidly established a peer-to-peer international network of laboratories to monitor changes in invasive bacterial diseases. We showed that in early 2020, the incidence of invasive disease due to *S pneumoniae, H influenzae*, and *N meningitidis* declined sharply in every country in the IRIS network, relative to rates in 2018 and 2019. These decreases corresponded with the timing of government responses to COVID-19, as measured by the OxCGRT, and changes in the movement of people, as measured by Google COVID-19 Community Mobility Reports.^17^ By contrast, the incidence of invasive disease due to *S agalactiae* (which is not transmitted by the respiratory route) in nine IRIS laboratories in 2020 did not differ significantly from the 2 previous years.

These IRIS data showed a significant reduction in invasive diseases caused by three bacterial pathogens that are, like SARS-CoV-2, transmitted via the respiratory route. The most plausible explanation for this observed reduction was the interruption of person-to-person bacterial respiratory transmission. Despite wide variations in the stringency of containment measures, the timing of these measures in all countries represented here coincided with a rapid reduction in the incidence of these invasive diseases. Mobility data suggest that people also voluntarily reduced their personal risks during the early stages of the pandemic. There might have also been a reduction in post-viral invasive bacterial diseases as a consequence of reductions in transmission of, and disease due to, respiratory viruses other than SARS-CoV-2.^18^, ^19^ We, therefore, contend that the IRIS data were a proxy for the effectiveness of public health measures undertaken to reduce the transmission of respiratory pathogens. The effect on bacterial transmission cannot be assumed to be identical to that on SARS-CoV-2, but it is likely to have followed a similar trajectory.

As the ecological niche of *S pneumoniae* is typically the nasopharynx of children, we also assessed the extent to which school closures explained the significant reduction in invasive diseases caused by *S pneumoniae*. Adding parameters based on the week of enforced school closure did not significantly improve fit over the model with parameters for workplace mobility data as a general measure of changed behaviours. Therefore, although school closures would have contributed to the observed reductions in movement of people and reduced transmission of *S pneumoniae*, in addition to physical distancing and other measures, closing schools was not associated with a detectable additional reduction in invasive pneumococcal disease. It is also possible that shielding of older adults, regardless of school closures, could have reduced bacterial transmission.^20^, ^21^, ^22^

Globally, morbidity and mortality rates associated with *S pneumoniae, H influenzae*, and *N meningitidis* are high. Safe and effective vaccines are available, and although these vaccines do not protect against all serotypes of each pathogen, they have been successfully implemented in childhood immunisation programmes in many countries. Nevertheless, vaccination is far from comprehensive and public health efforts must remain focused on these three pathogens.^23^, ^24^, ^25^, ^26^ Any disruptions to existing vaccination programmes as a result of the pandemic also need to be urgently addressed.^27^, ^28^ Furthermore, in the context of preventing transmission, the current *S pneumoniae, H influenzae* serotype b, and *N meningitidis* conjugate-polysaccharide vaccines are successful in large part because they induce herd immunity by reducing bacterial colonisation and thereby interrupt transmission. These IRIS data underline the importance of reducing person-to-person transmission of respiratory pathogens.^29^

Our study highlights the crucial importance of active microbiological surveillance for public health and the fundamental role of reference laboratories. Surveillance is most effective when it is done consistently, provides high-quality data, and continues uninterrupted for many years so that emerging trends can be detected with confidence. Here, we have compared data for 2020 with data for 2018 and 2019 because the rates of disease were markedly altered in 2020, but most IRIS laboratories have been undertaking high-quality surveillance for many years, or even several decades.

The strengths of the IRIS Initiative included the formation of a network of well established and experienced national laboratory and surveillance programmes, which rapidly provided high-quality invasive bacterial disease data. The IRIS laboratories are leading comprehensive and sustained surveillance programmes that have been systematically collecting and testing microbiological data and samples for many years. The IRIS network includes 26 countries and territories across six continents and a large overall dataset. The consistency in the trends observed across all individual datasets provided confidence in our interpretations. The IRIS Initiative was established through existing international public health and academic networks in response to COVID-19, and the network of collaborators and the data produced will be an invaluable resource to observe and investigate future changes. Additional disease perturbations are to be expected as COVID-19-related containment measures are modified, which will allow greater insight into the effects of specific public health interventions. The IRIS laboratories will have a central role in rapidly detecting further invasive disease perturbations in their respective countries and territories.

Uniquely, we analysed invasive bacterial disease data, OxCGRT indices, and Google COVID-19 Community Mobility Reports data per country, which provided the means to assess the associations between country-specific containment measures, changes in the movements of human populations, and the corresponding reductions in the incidence of invasive diseases.

Potential limitations in these analyses include the incompleteness of the submissions to individual laboratories, which would mitigate the observed reduction in invasive diseases. However, in most participating countries, invasive diseases due to these pathogens are legally notifiable and submissions are made routinely by well established systems, often to guide diagnosis or public health action, all of which continued throughout this period. Importantly, numbers of submissions of invasive *S agalactiae* cases over the same period were as expected. Furthermore, although we could not completely exclude the possibility that people with these invasive diseases were less likely to seek health care because of the pandemic, given that invasive disease is a medical emergency and the data presented here were based on bacteria detected from clinical specimens taken in hospital (eg, blood and cerebrospinal fluid), the significant and sustained reductions in bacterial invasive disease reported in every country were unlikely to be due to reduced hospital admissions. Invasive meningococcal isolates were reported to be variably affected by COVID-19 lockdown measures in France, depending on their phenotypes and genotypes.^16^ These data suggest that highly invasive and highly transmitted meningococci were affected by lockdown to a greater extent than other meningococci. These French data therefore also support the hypothesis that reduced transmission rates were due to COVID-19 containment measures and not underreporting, which would be expected to affect all isolates in a similar manner.

Another potential limitation of our study is that the countries participating in IRIS are high-income and middle-income countries, which might reduce the generalisability of our results; however, a key message from these data is that reducing respiratory transmission of bacteria will reduce the incidence of invasive disease, which will be beneficial in any country. Low-income countries have the highest global burden of invasive diseases and the benefits of reducing transmission might therefore be even greater than for middle-income and high-income countries. Lastly, some changes that could reduce microbial transmission are less easily measured and could, therefore, not be accounted for, such as parents keeping their children out of school because of fear of infection before schools were officially closed. These difficult-to-measure factors could have led to an underestimation of the effect of containment measures on reductions in disease transmission.

SARS-CoV-2 has provided a stark reminder that infectious diseases are a major threat to the lives and livelihoods of people worldwide. Although the COVID-19 pandemic has resulted in drastic containment measures in many countries, highly restrictive measures are unsustainable in the longer term. However, straightforward measures (eg, frequent handwashing) should be routine behaviour within every population to reduce pathogen transmission. The prevention of disease through vaccination is also crucial. The COVID-19 pandemic has revealed opportunities to accelerate public health advances in medicine and technology. Although COVID-19 is the most prominent current infectious disease challenge, others remain and other pathogens will emerge in the future. This study provides an example of how the international public health and scientific community can collaborate to apply knowledge, experience, and data analyses to improve global health.

## Data sharing

It is not possible to share the study data because doing so would risk identifying individual cases of invasive disease in some countries; however, source code for the analyses is available via GitHub: https://github.com/brueggemann-lab/iris-ldh-2020.

## Declaration of interests

The following authors received support for work unrelated to this study: MPGvdL has received grants from Pfizer, Merck, and the Robert Koch Institut; RB has done contract research on behalf of Public Health England for GlaxoSmithKline, Pfizer, and Sanofi Pasteur, but received no personal remuneration; MC has received grants from Pfizer; SAC has done contract research on behalf of Public Health England for GlaxoSmithKline, Pfizer, and Sanofi Pasteur, but received no personal remuneration; SD has received a grant from Pfizer; SJG did contract research (carriage studies) for vaccine manufacturers (GlaxoSmithKline and Pfizer) on behalf of Public Health England, but received no personal remuneration; MH has received grants from Pfizer and the Federal Office of Public Health, and personal fees (for being on an advisory board) from Pfizer and Merck Sharp & Dohme; HH has received grants from Astellas and Pfizer; KAJ has received a grant from Wellcome Trust and personal fees from GlaxoSmithKline; SNL has done contract research for vaccine manufacturers (GlaxoSmithKline, Pfizer, and Sanofi Pasteur) on behalf of St. George's University of London, but received no personal remuneration; DJL has received grants from GlaxoSmithKline and Pfizer; SM has received a grant from Sanofi Pasteur; CM-A has received grants from Quiastat, Roche, Pfizer, and Genomica, and personal fees from Roche, Pfizer, and Qiagen; LS has received a grant from GlaxoSmithKline; H-CS has received a grant from Pfizer; MI has received non-financial support from GlaxoSmithKline and Pfizer, personal fees from Pfizer (speaker fees) and Merck Sharp & Dohme (speaker fees), and grants from Merck Sharp & Dohme; M-KT has received grants from GlaxoSmithKline, Pfizer, and Sanofi Pasteur; ASk has received grants and non-financial support from Pfizer, and personal fees from Pfizer, Merck Sharp & Dohme, and Sanofi Pasteur; CLS has received grants from Pfizer and GlaxoSmithKline for investigator-led research; EV has received grants on behalf of her institution (Intercommunal Hospital of Créteil) from Pfizer and Merck Sharp & Dohme; MT has received grants from GlaxoSmithKline and Pfizer; NKF's institution (Public Health England) has received funding for investigator-initiated research from GlaxoSmithKline, Pfizer, and other vaccine manufacturers (GlaxoSmithKline, Pfizer, and Affinivax), but NKF received no personal remuneration; AvG has received a grant from Sanofi Pasteur; NMvS has received a grant from Pfizer, a fee for service paid to their institution from Merck Sharp & Dohme and GlaxoSmithKline, and also has a patent (WO 2013/020090 A3) on vaccine development against Streptococcus pyogenes, unrelated to this study, with royalties paid to University of California San Diego, CA, USA; and MKT has a patent (630133) for a vaccine for serogroup X meningococcus with GlaxoSmithKline. All other authors declare no competing interests.
