# Correction to *Lancet Digit Health* 2021; 3: e360–70

**DOI:** 10.1016/S2589-7500(21)00103-5

**Published:** 2021-05-26

*Brueggemann AB, Jansen van Rensburg MJ, Shaw D, et al. Changes in the incidence of invasive disease due to* Streptococcus pneumoniae, Haemophilus influenzae*, and* Neisseria meningitidis *during the COVID-19 pandemic in 26 countries and territories in the Invasive Respiratory Infection Surveillance Initiative: a prospective analysis of surveillance data*. Lancet Digit Health *2021;* 3: *e360–70*—In the Summary and Results sections of this Article, the total number of *Streptococcus pneumoniae* has been corrected to 62 434 following the removal of 403 duplicate isolate *S pneumoniae* records for Denmark for the years 2018 and 2019. Data changes to figure 1–3 have also been made following the removal of these duplicate data. The removal of these data did not affect the statistical analysis. The appendix has also been corrected. These corrections have been made as of May 26, 2021.

