# Supplementary material for: Changes in the incidence of invasive disease due to Streptococcus pneumoniae, Haemophilus influenzae, and Neisseria meningitidis during the COVID-19 pandemic in 26 countries and territories in the Invasive Respiratory Infection Surveillance Initiative: a prospective analysis of surveillance data
Source: Lancet Digit Health. 2021 May 24;3(6):e360–70. doi: 10.1016/S2589-7500(21)00077-7 (PMC8166576; doi:10.1016/S2589-7500(21)00077-7)
Supplement: Supplementary appendix [file mmc1.pdf]

THE LANCET
Digital Health

Supplementary appendix

This appendix formed part of the original submission and has been peer reviewed. We post it as supplied by the authors.

Supplement to: Brueggemann AB, Jansen van Rensburg MJ, Shaw D, et al. Changes in the incidence of invasive disease due to *Streptococcus pneumoniae*, *Haemophilus influenzae*, and *Neisseria meningitidis* during the COVID-19 pandemic in 26 countries and territories in the Invasive Respiratory Infection Surveillance Initiative: a prospective analysis of surveillance data. *Lancet Digit Health* 2021; **3:** e360–70.

Supplementary Material

Contents

Supplementary Tables 2

Supplementary Table 1. Laboratories participating in the Invasive Respiratory Infection Surveillance (IRIS) initiative 2

Supplementary Table 2. Country-specific weekly interruption time points used in the time series analyses. .. 4

Supplementary Methods 5

Equation for individual country models 5

Supplementary Figures 6

Supplementary Figure 1. Interrupted time series modelling to quantify changes in the incidence of invasive disease due to four pathogens. 6

Supplementary Figure 2. Annual invasive *H influenzae* cases submitted to Invasive Respiratory Infection Surveillance laboratories in 24 countries and territories from Jan 1, 2018, to May 31, 2020. 7

Supplementary Figure 3. Annual invasive *N meningitidis* cases submitted to Invasive Respiratory Infection Surveillance laboratories in 21 countries and territories from Jan 1, 2018, to May 31, 2020. 8

Supplementary Figure 4. Annual invasive *S agalactiae* cases submitted to Invasive Respiratory Infection Surveillance laboratories in nine countries from Jan 1, 2018, to May 31, 2020. 9

Supplementary Figure 5. Assessment of the movement of people in Invasive Respiratory Infection Surveillance (IRIS) countries using Google COVID-19 Community Mobility Reports (CCMR) data. 10

Supplementary Tables

Supplementary Table 1. Laboratories participating in the Invasive Respiratory Infection Surveillance (IRIS) initiative

Country	Laboratory
Belgium	National Reference Centre for *Haemophilus influenzae*, Laboratoires des Hôpitaux Universitaires de Bruxelles - Universitaire Laboratorium Brussel, Brussels, Belgium; Faculté de Médecine et Pharmacie, Université de Mons, Mons, Belgium
	National Reference Centre for *Neisseria meningitidis*, Sciensano, Brussels, Belgium
	National Reference Centre for *Streptococcus pneumoniae*, University Hospitals Leuven, Leuven, Belgium; Department of Microbiology, Immunology and Transplantation, KU Leuven, Leuven, Belgium
Brazil	National Laboratory for Meningitis and Pneumococcal Infections, Center of Bacteriology, Institute Adolfo Lutz, São Paulo, Brazil
Canada	National Microbiology Laboratory, Public Health Agency of Canada, Winnipeg, Manitoba, Canada
China	Department of Pulmonary and Critical Care Medicine, Center of Respiratory Medicine, National Clinical Research Center for Respiratory Diseases, Institute of Respiratory Medicine, Chinese Academy of Medical Sciences, Peking Union Medical College, Beijing, China
Czech Republic	National Reference Laboratory for Haemophilus Infections, Centre for Epidemiology and Microbiology, National Institute of Public Health, Prague, Czech Republic
	National Reference Laboratory for Meningococcal Infections, Centre for Epidemiology and Microbiology, National Institute of Public Health, Prague, Czech Republic
	National Reference Laboratory for Streptococcal Infections, Centre for Epidemiology and Microbiology, National Institute of Public Health, Prague, Czech Republic
Denmark	Department of Bacteria, Parasites and Fungi, Statens Serum Institut, Copenhagen, Denmark
England	Meningococcal Reference Unit, National Infection Service, Public Health England, Manchester Royal Infirmary, Manchester, United Kingdom
	Respiratory and Vaccine Preventable Bacteria Reference Unit, National Infection Service, Public Health England, London, United Kingdom
Finland	Finnish Institute for Health and Welfare (THL), Helsinki, Finland
France	Institut Pasteur, Invasive Bacterial Infections Unit and National Reference Centre for Meningococci and Haemophilus influenzae, Paris, France
	Laboratory of Medical Biology and National Reference Centre for Pneumococci, Intercommunal Hospital of Créteil, Créteil, France
Germany	German National Reference Center for Meningococci and *Haemophilus influenzae*, Institute for Hygiene and Microbiology, University of Würzburg, Würzburg, Germany
	German National Reference Center for Streptococci, Department of Medical Microbiology, University Hospital RWTH Aachen, Aachen, Germany
Hong Kong Special Administrative Region of the People's Republic of China	Department of Microbiology, The Chinese University of Hong Kong, Hong Kong
	Microbiology Division, Public Health Laboratory Services Branch, Centre for Health Protection, Department of Health, Hong Kong
Iceland	Department of Clinical Microbiology, Landspitali, The National University Hospital of Iceland, Reykjavik, Iceland
Ireland	Irish Meningitis and Sepsis Reference Laboratory, Children's Health Ireland at Temple Street, Dublin, Ireland
Israel	Government Central Laboratories, Ministry of Health, Jerusalem, Israel
Luxembourg	Laboratoire National de Sante, Dudelange, Luxembourg
Netherlands	Department of Medical Microbiology and Infection Prevention and Netherlands Reference Laboratory for Bacterial Meningitis, Amsterdam University Medical Center, University of Amsterdam, Amsterdam, Netherlands
New Zealand	Meningococcal Reference Laboratory, Institute of Environmental Science and Research Limited, Porirua, New Zealand

Country	Laboratory
	Streptococcal Reference Laboratory, Institute of Environmental Science and Research Limited, Porirua, New Zealand
Northern Ireland	Public Health Agency, Belfast, Northern Ireland
Poland	National Reference Centre for Bacterial Meningitis, National Medicines Institute, Warsaw, Poland
Scotland	Bacterial Respiratory Infection Service, Scottish Microbiology Reference Laboratories, Glasgow, Scotland, United Kingdom
South Africa	Centre for Respiratory Diseases and Meningitis, National Institute for Communicable Diseases, Division of the National Health Laboratory Service, Johannesburg, South Africa
South Korea	Department of Pediatrics, Seoul National University College of Medicine, Seoul, South Korea
	Division of Infectious Diseases, Department of Internal Medicine, Korea University Guro Hospital, Korea University College of Medicine, Seoul, South Korea
Spain	Instituto de Recerca Pediatrica, Hospital Sant Joan de Deu, Barcelona, Spain
Sweden	Department of Clinical Microbiology, Karolinska University Hospital, Stockholm, Sweden
	National Reference Laboratory for *Neisseria meningitidis*, Department of Laboratory Medicine, Clinical Microbiology, Faculty of Medicine and Health, Örebro University, Örebro, Sweden
Switzerland	Swiss National Reference Centre for invasive Pneumococci, Institute for Infectious Diseases, University of Bern, Bern, Switzerland
Wales	Public Health Wales, Cardiff, Wales, United Kingdom

Supplementary Table 2. Country-specific weekly interruption time points used in the time series analyses.

Country	Week of the year 2020[a]
Belgium	12
Brazil	12
Canada	12
China[b]	5
Czech Republic	11
Denmark	11
England	12
Finland	12
France	11
Germany	12
Hong Kong Special Administrative Region of the People's Republic of China	7
Iceland[b]	11
Ireland	12
Israel	11
Luxembourg	11
Netherlands	11
New Zealand	13
Northern Ireland	12
Poland	11
Scotland	12
South Africa	12
South Korea	8
Spain	11
Sweden	11
Switzerland	11
Wales	12

[a] The chosen week for each country was based on the Google COVID-19 Community Mobility Reports (CCMR) data (see Figure 3 in the main text), selecting the week containing the midpoint of the decline in work-associated mobility.

[b] No Google CCMR data were available for China (due to censorship of Google data) and Iceland (the small national population presents a possible privacy breach). Iceland was assigned the modal week of other European countries, while China's interruption point was based on news reports of policy decisions and set at week 5 of 2020.

Supplementary Methods

Equation for individual country models

$\log(\text{Count}_t)=\text{Intercept} + \beta 1(T_t) + \beta 2(\text{sine}(2\pi*(W_t)/52) + \beta 3(\text{cosine}(2\pi*(W_t)/52) + \beta 4(\text{sine}(\pi*(W_t)/52) + \beta 5(\text{cosine}(\pi*(W_t)/52) + \beta 6(St_t) + \beta 7(Sl_t) + \varepsilon_t$ (Distribution Poisson, Scale 2)

Count_t = Cases in week t
T = time in weeks starting at week 1 2018
W = week of year
St= binary step change variable coded zero before COVID and 1 after COVID (with this change derived from the midpoint of the drop in mobility from Google data or equivalent)
Sl = slope change variable counting each week from the after the step change week
ε = Error term following Poisson Distribution Poisson with scale 2

Supplementary Figures

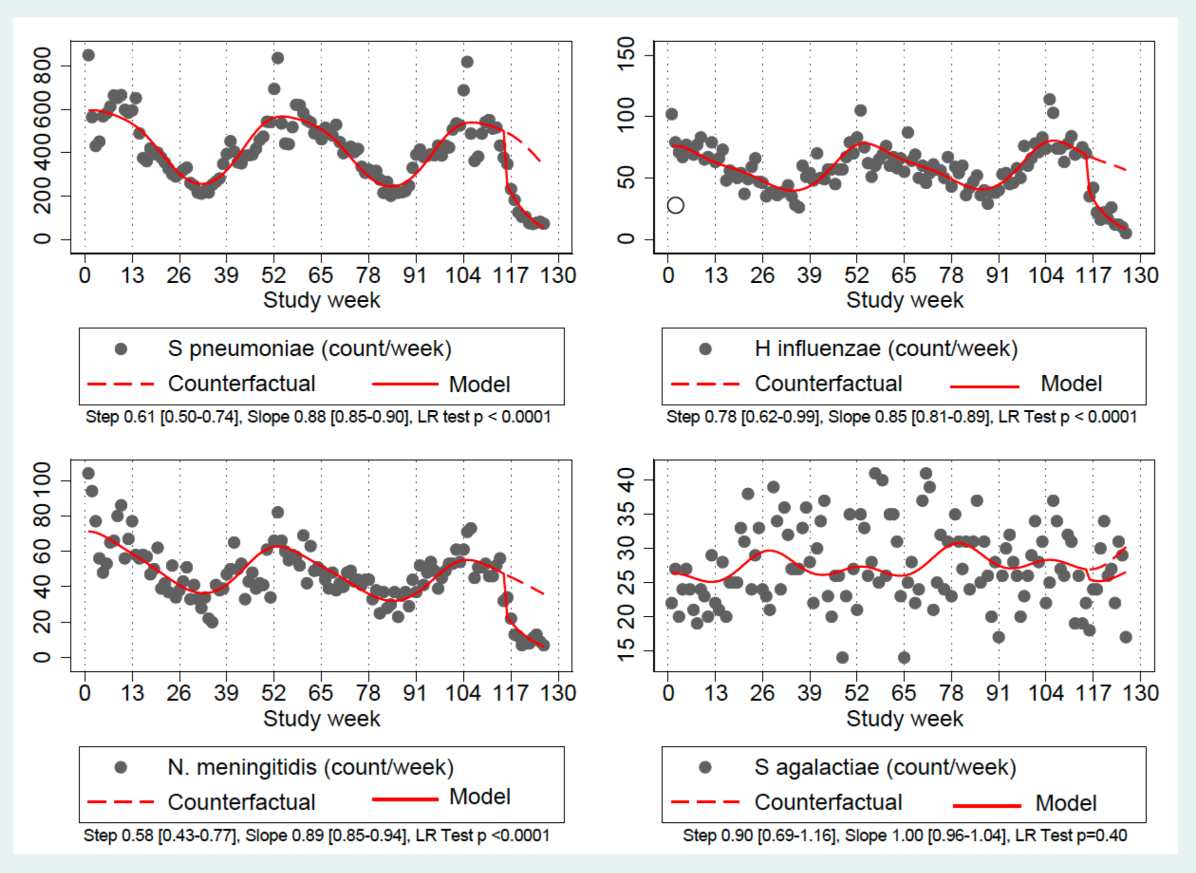

Supplementary Figure 1. Interrupted time series modelling to quantify changes in the incidence of invasive disease due to four pathogens. Observed counts per week are shown over the time period 1 January 2018 through 31 May 2020, a fitted model allowing for a step and slope change following week 11 of 2020 (study week 115), and a counterfactual model without this change for each species. Estimates for the step and slope (change per week) parameters and confidence intervals are given for each species, and a p-value from a likelihood ratio comparing models with both step and slope variables and models with neither.

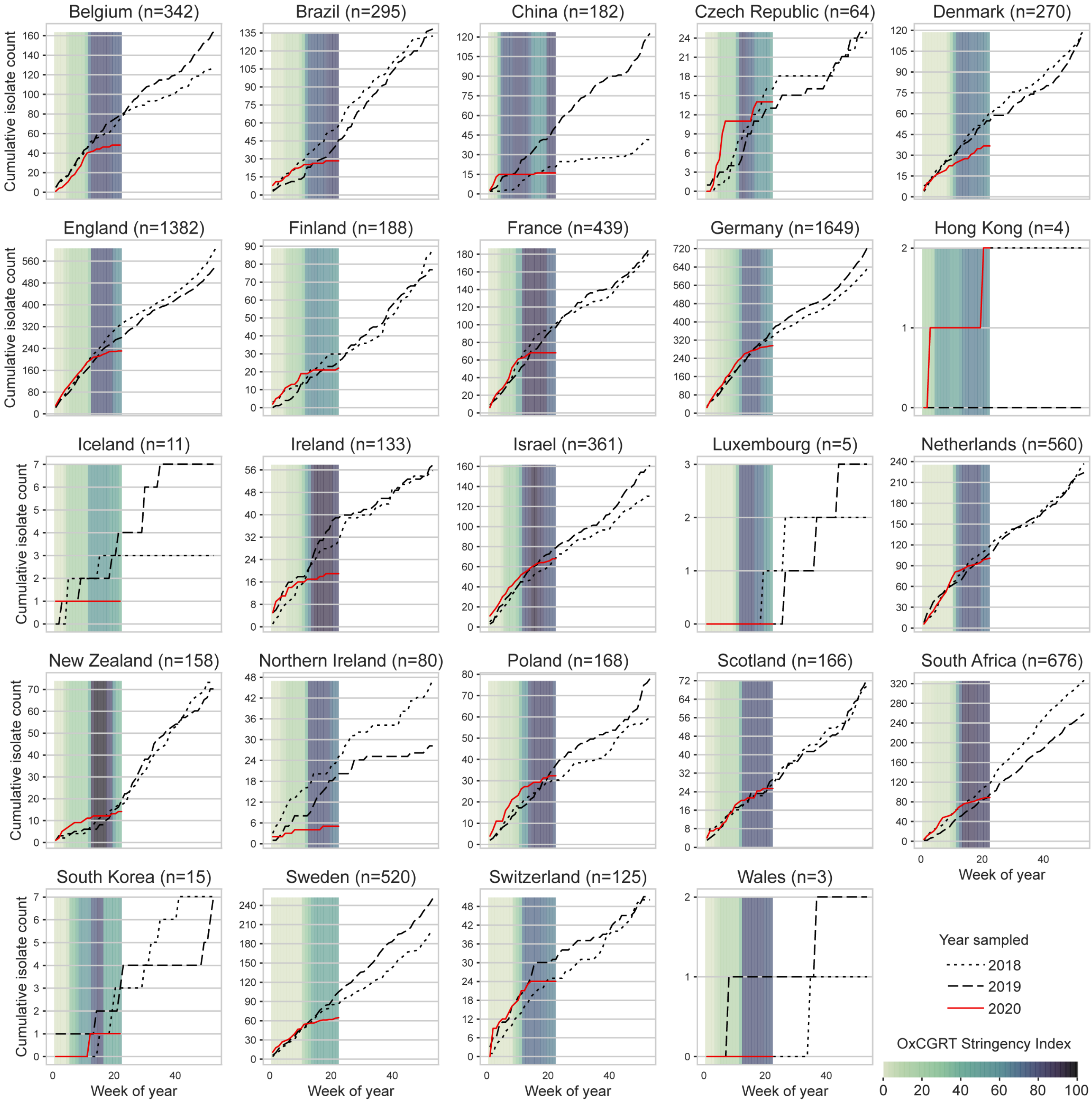

Supplementary Figure 2. Annual invasive *H influenzae* cases submitted to Invasive Respiratory Infection Surveillance laboratories in 24 countries and territories from Jan 1, 2018, to May 31, 2020. Coloured bars represent the mean weekly Oxford COVID-19 Government Response Tracker (OxCGRT) stringency index values on a scale from 0-100. Larger (darker) values indicate that higher stringency measures were enacted within a country. Data for South Korea were submitted from two surveillance networks, one of which started invasive disease surveillance in September, 2018, so data presented here for that hospital are only from September, 2018, onwards, whereas the data from the other hospital are from January, 2018, onwards.

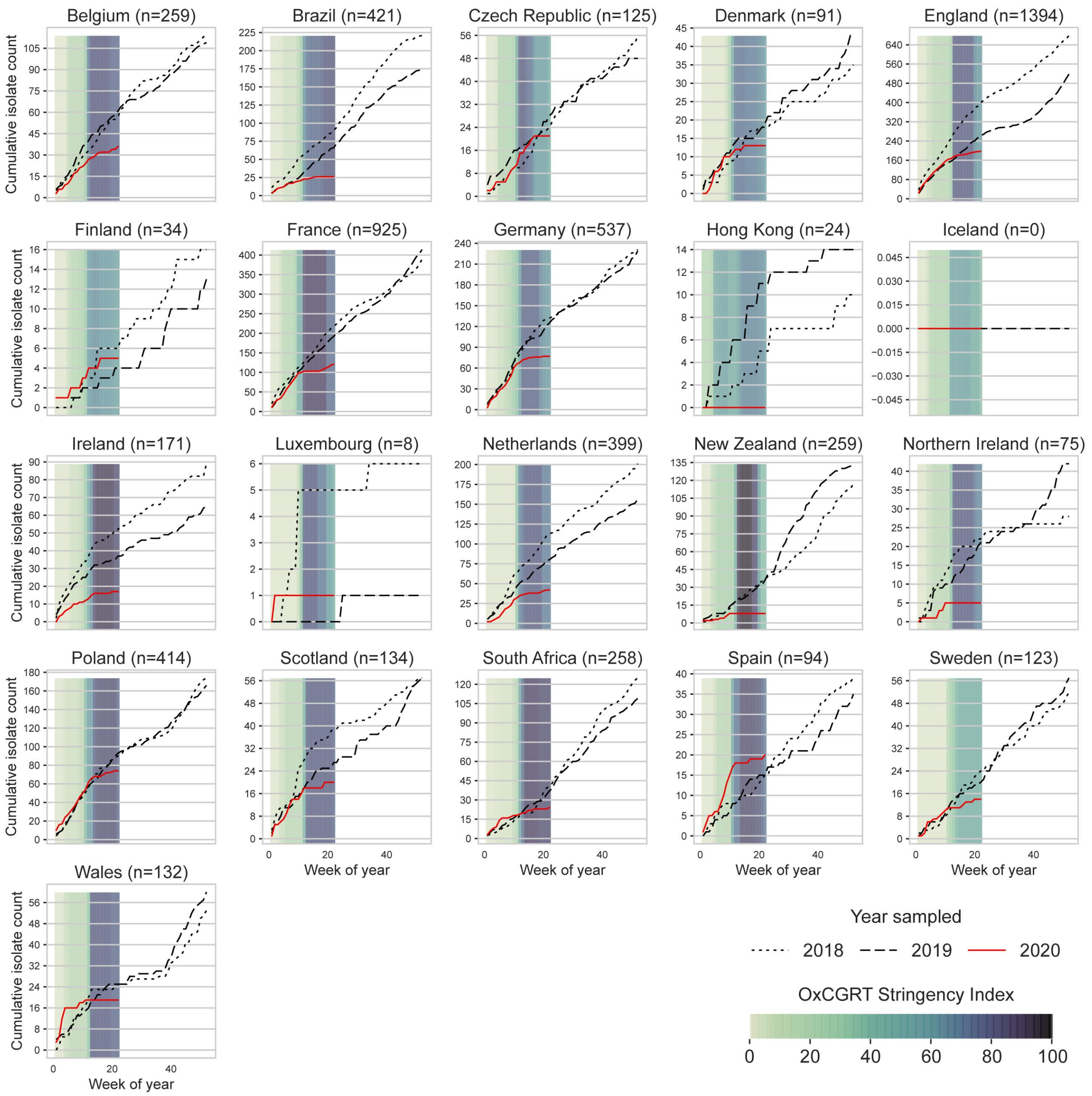

Supplementary Figure 3. Annual invasive *N meningitidis* cases submitted to Invasive Respiratory Infection Surveillance laboratories in 21 countries and territories from Jan 1, 2018, to May 31, 2020. Coloured bars represent the mean weekly Oxford COVID-19 Government Response Tracker (OxCGRT) stringency index values on a scale from 0-100. Larger (darker) values indicate that higher stringency measures were enacted within a country.

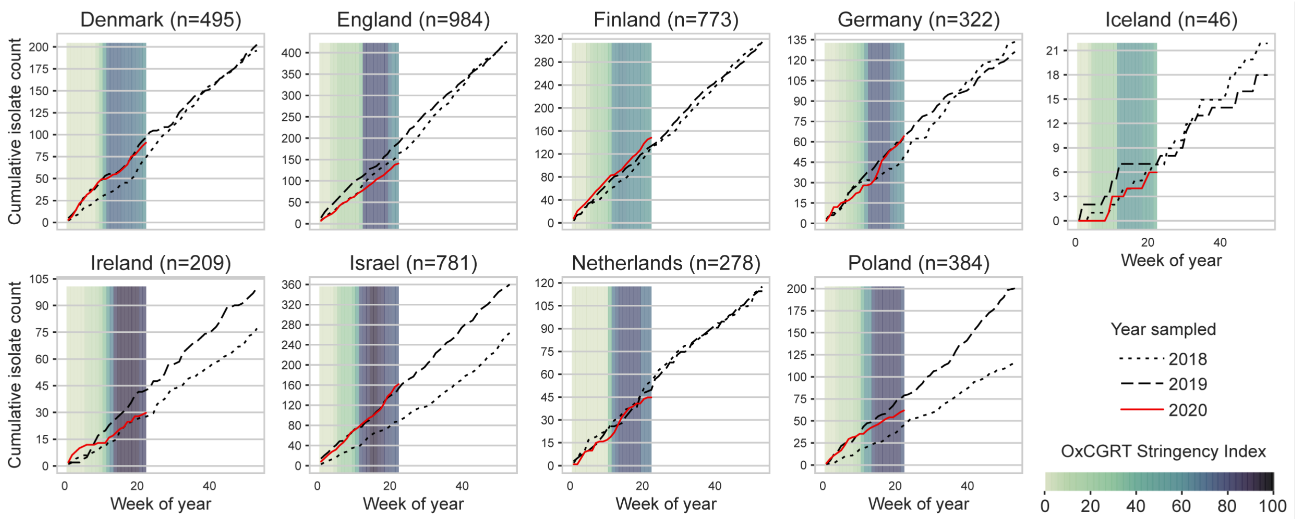

Supplementary Figure 4. Annual invasive *S agalactiae* cases submitted to Invasive Respiratory Infection Surveillance laboratories in nine countries from Jan 1, 2018, to May 31, 2020. Coloured bars represent the mean weekly Oxford COVID-19 Government Response Tracker (OxCGRT) stringency index values on a scale from 0-100. Larger (darker) values indicate that higher stringency measures were enacted within a country.

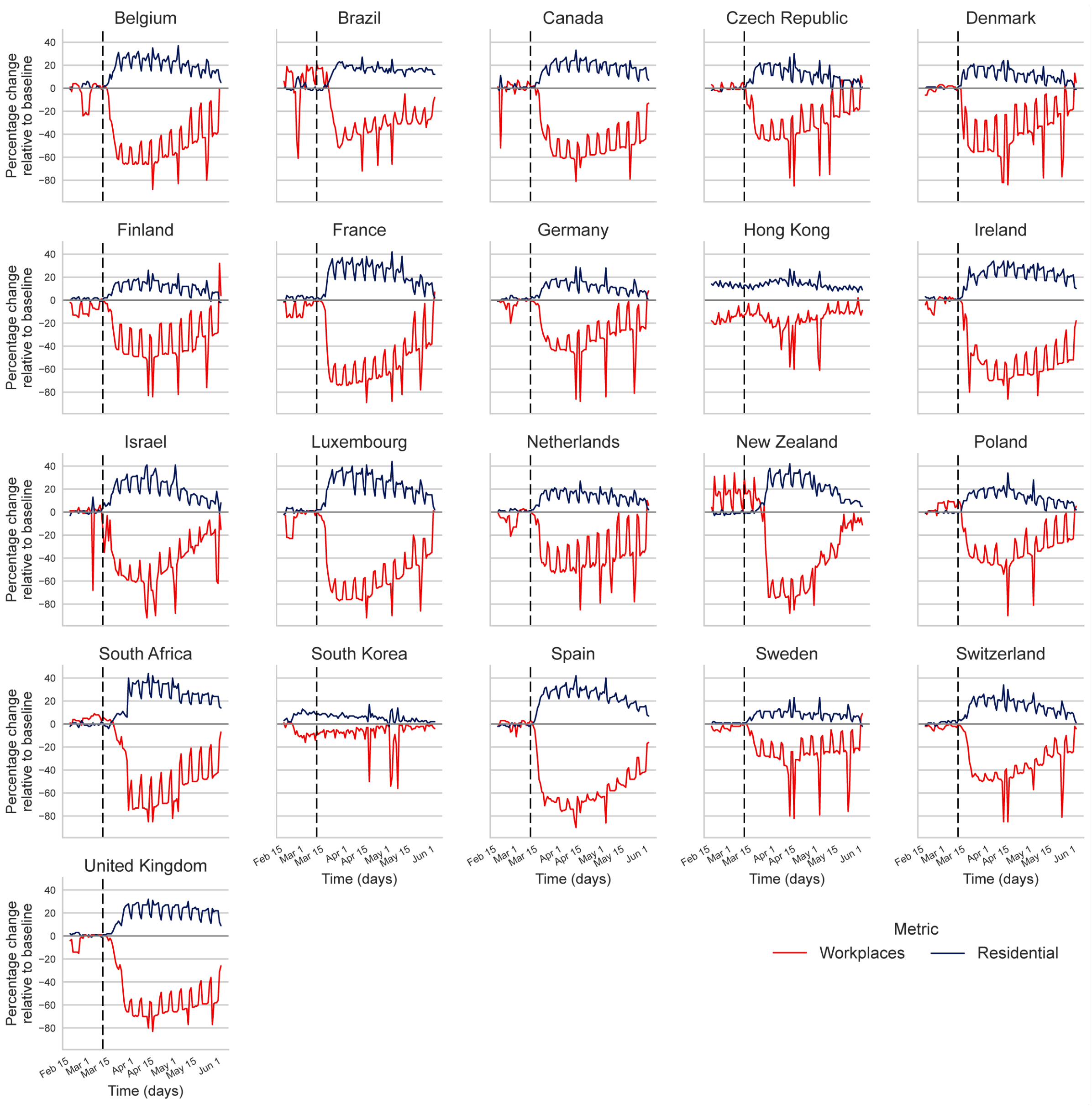

Supplementary Figure 5. Assessment of the movement of people in Invasive Respiratory Infection Surveillance (IRIS) countries using Google COVID-19 Community Mobility Reports (CCMR) data. Workplaces and Residential data are plotted for each country participating in IRIS except for China (due to censorship of Google data) and Iceland (the small national population presents a possible privacy breach). Google CCMR data for the United Kingdom aggregated England, Scotland, Wales, and Northern Ireland as one dataset. The dashed vertical line marks week 11, when the WHO officially declared the COVID-19 pandemic. The periodic nature of the graphs is because these are daily data, and the movement of people changes at the weekends; the sharp data spikes typically represent national holidays such as the Easter weekend in mid-April.
